# Laparoscopy-Assisted Percutaneous Nephrolithotripsy (PCNL) in Ectopic Pelvic Kidney

**DOI:** 10.7759/cureus.26928

**Published:** 2022-07-16

**Authors:** Abdolsalam Ahmadi, Ahmed A Al Rashed, Sayed H Ebrahim, Omran Hasan, Husain Alaradi, Khalid Abdulaziz, Akbar Jalal, Nader Awad

**Affiliations:** 1 Urology, Salmaniya Medical Complex, Manama, BHR; 2 Surgery, Salmaniya Medical Complex, Manama, BHR

**Keywords:** percutaneous nephrolithotomy (pcnl), recurrent nephrolithiasis, staghorn stone, pelvic ectopic kidney, laparoscopic assisted pcnl

## Abstract

An ectopic kidney is a rare developmental anomaly in which the kidney can be pelvic, iliac, abdominal, and thoracic, and affected patients are more prone to conditions such as reflux, pelvic ureteric junction (PUJ) obstruction, hydronephrosis, nephrolithiasis, and even renal failure than patients with normally structured kidneys. In this case, we present a 43-year-old male who is a known case of ectopic left pelvic kidney and presented with chronic lower abdominal pain. Upon imaging, it was revealed that he had a staghorn stone for which he underwent laparoscopy-assisted percutaneous nephrolithotripsy (PCNL).

Postoperatively, the patient underwent a quick recovery and was discharged on postoperative day 3 without any perioperative complications. Hence given our experience with this case and the similar experiences of urologists over time, the use of laparoscopy-assisted PCNL appears to create a safe way of entering the abdomen and locating the ectopic kidney as well as provide visual guidance in puncture and dilatation all the while protecting the adjacent structures from harm. This demonstrates that laparoscopy-assisted PCNL is a feasible safe and minimally invasive procedure for patients with ectopic kidneys presenting with large stones.

## Introduction

An ectopic kidney is a rare developmental anomaly with postmortem and clinical incidence suggesting 1:900 and 1:12,000, respectively [1]. The ectopic kidney’s location can be pelvic, iliac, abdominal, or thoracic. It can present as unilateral or crossed/crossed fused, a condition known as crossed renal ectopia [2]. Due to the abnormal and aberrant rotation of an ectopic pelvic kidney, affected patients are more prone to conditions such as reflux, hydronephrosis, nephrolithiasis, and even renal failure than patients with normally structured kidneys [3].

In contrast to the normally structured kidneys, the management of nephrolithiasis in ectopic kidneys differs and there are no clear consensuses to the optimum approach [4]. Percutaneous nephrolithotripsy (PCNL) has been regarded as the favored modality in the treatment of stones that are larger than 2 cm in diameter [5]. However, this approach could carry a risk of injury to the surrounding vascular structures or adjacent organs, particularly the overlying bowels [5] when dealing with ectopic kidneys. Thus, the use of a laparoscope to assist in the procedure provides a safe way of clearing the stone herein making this procedure a favored minimally invasive intervention for such cases.

Here, we present a case of a 43-year-old male who was known to have an ectopic left pelvic kidney and presented with a staghorn stone, and underwent laparoscopy-assisted PCNL.

## Case presentation

**Figure 1:**
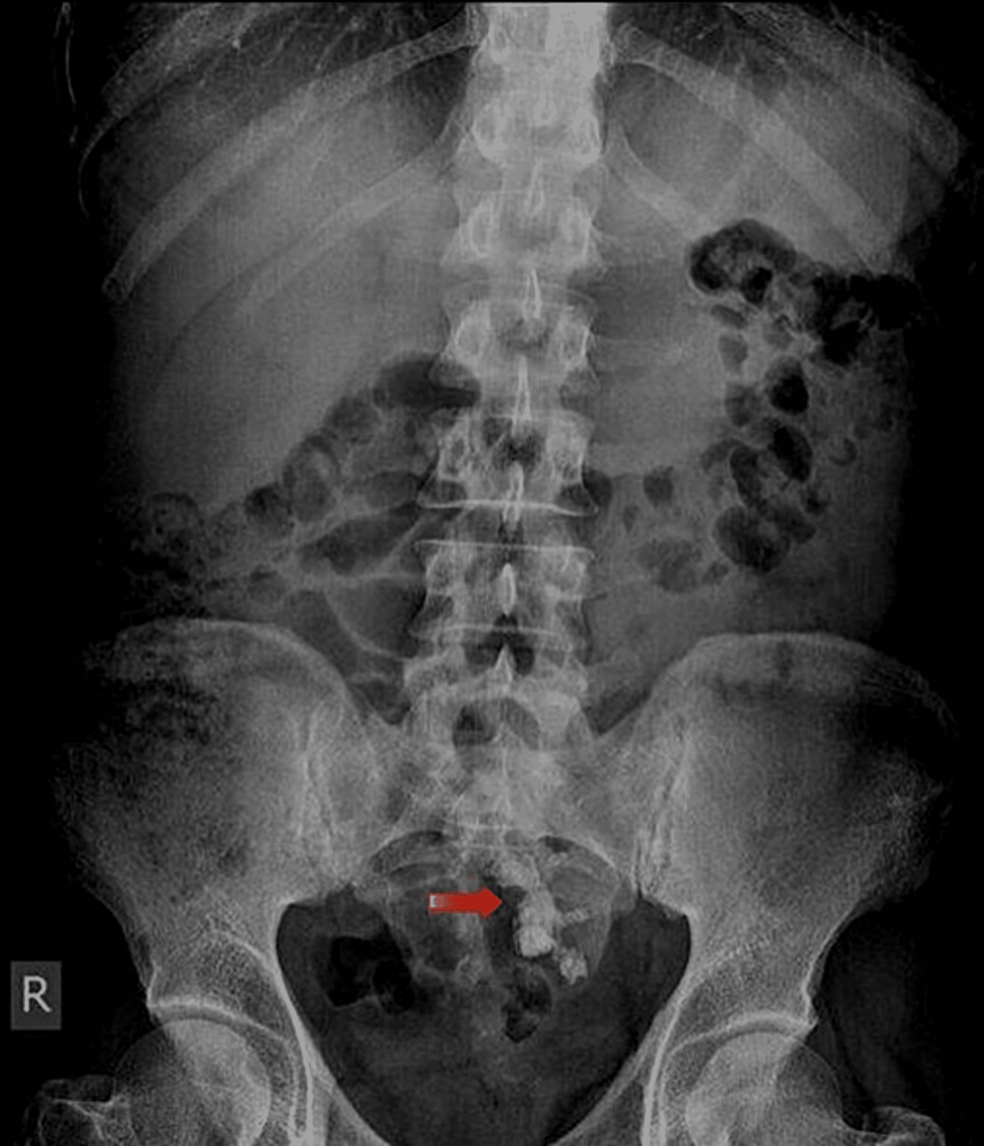
KUB x-ray demonstrating a shadow of a staghorn stone in the pelvic region KUB - Kidney Ureter Bladder

A 43-year-old male with a left pelvic kidney and a history of chronic left lower abdominal pain that has been bothersome for the past five years' duration. He reported the pain to be colicky in nature and associated with intermittent episodes of gross hematuria. His past surgical history included multiple attempted ureteroscopy procedures at other facilities on the left kidney for nephrolithiasis. Initial investigative workup showed microscopic hematuria (RBC > 100 cells/HPF) on urine analysis with no concurrent urinary tract infection and full blood count (Hb 14.5 g/dL) and renal function were noted to be normal (creatinine 67 µmol/L). A kidney ureter bladder (KUB) x-ray was performed and demonstrated a large radio-opaque shadow in the pelvis most likely representing the staghorn stone located within the ectopic left kidney (Figure 1).

**Figure 2:**
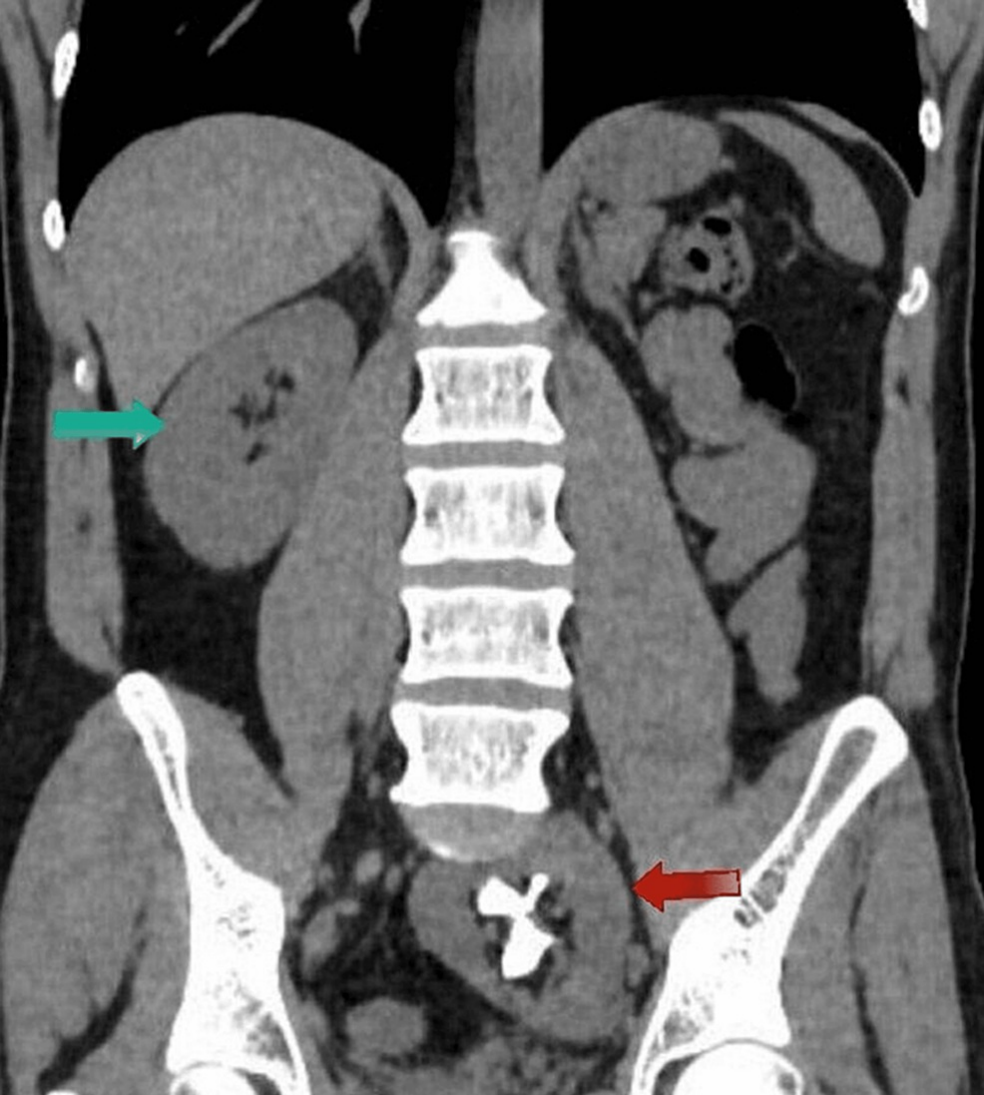
Coronal view CT abdomen showing left pelvic kidney with staghorn stone (red arrow) and normally located right kidney (green arrow)

Furthermore, prior to surgical intervention, a computerized tomography (CT) of the abdomen was done pre and post-contrast imaging and illustrated an ectopic left kidney located in the left hemipelvis with a malrotated hilum facing anteriorly. Moreover, a large (3.5cm by 2.5cm) staghorn calculus conforming to the shape of the collecting system was noted within the left kidney as seen in Figure 2 with no significant hydroureter or hydronephrosis and no evidence of pyelonephritis.

Additionally, there was an incidental finding of a 2-mm nonobstructive calculus in the lower pole of the right kidney along with an upper pole 5mm right renal cyst. Following confirmation of the stone size and location with the CT scan, the condition was explained to the patient and he underwent a laparoscopy-assisted PCNL. 

Intra-operatively, initial cystoscopy was performed followed by a left retrograde study, once the anatomy was identified, a ureteric catheter was kept in place. Furthermore, two trocar site incisions were done, a 12-mm supraumbilical incision followed by a 5-mm right iliac fossa incision, and trocars were inserted followed by inserting the laparoscopic camera through the right iliac fossa trocar and locating the pelvic kidney through a transperitoneal approach. Following that, the bowel loops overlying the pelvic kidney were cleared, this was done through bowel mobilization using Maryland forceps followed by positioning the patient's head down in order to retract the bowel away from the kidney. A Chiba needle was inserted under combined fluoroscopic and laparoscopic guidance into the renal mid-calyx through which dilatation was done under vision. Once dilated 24 Fr Amplatz sheath was fixed and a 20 Fr Nephroscope was introduced. Subsequently, the stone was dusted and sucked using Swiss Ultrasonic Lithoclast until 100% clearance was achieved and finally a double J stent was kept in place. Finally, an intra-abdominal drain was placed.

**Figure 3:**
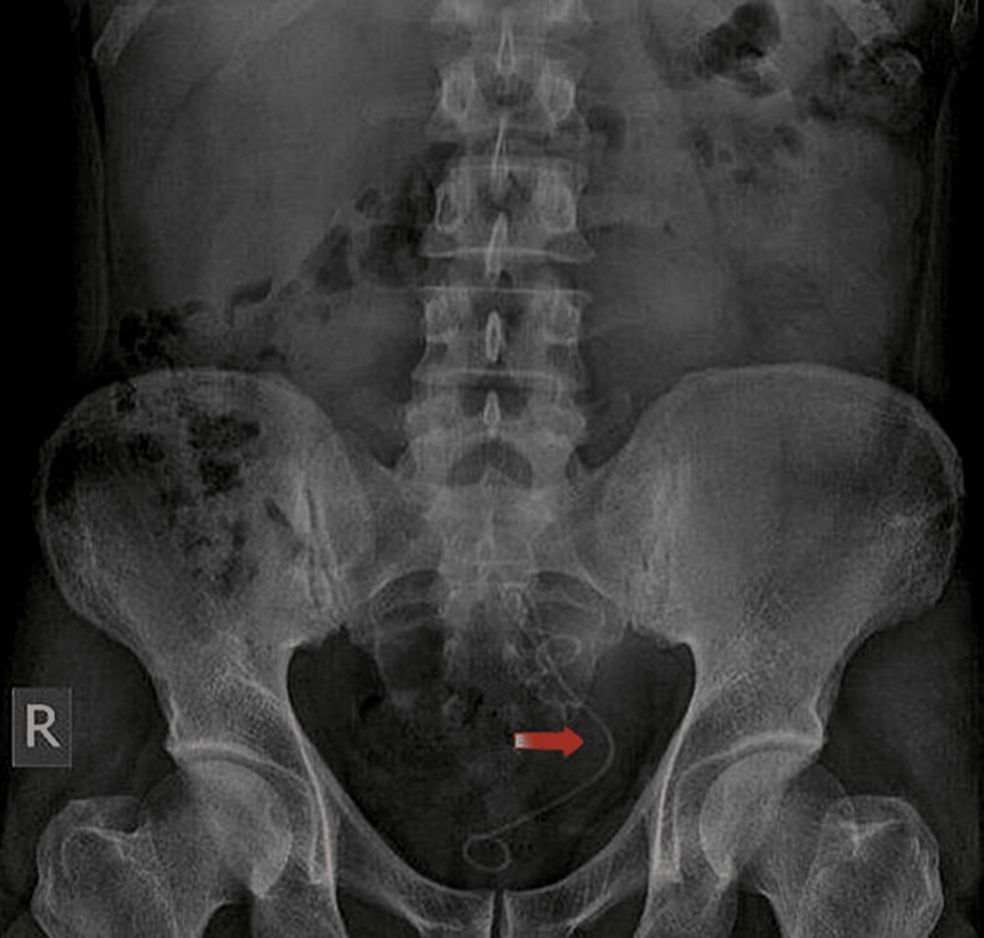
Post-op KUB x-ray showing left double J stent in place with no evidence of residual stone KUB - Kidney Ureter Bladder

Postoperatively, the patient underwent a quick recovery and foleys catheter was removed on the first postoperative day. A post operative KUB x-ray showed double J stent in place in the left pelvic kidney with complete clearance of the previously noted stone (Figure 3).

Furthermore, the drain was removed on the second postoperative day and the patient was discharged in a stable condition. Finally, he was followed up in the outpatient department after two weeks and was doing well. Currently, he is scheduled for removal of double J stent at one-month postoperatively as a day case procedure.

## Discussion

When the kidney is atypically placed due to the faulty migration from the kidney during embryological development, this is known as renal ectopia [6]. One of the most common complications that accompany ectopic kidneys is the development of nephrolithiasis [6] and the management of calculi in such cases poses different challenges to the surgeon depending on the location of the kidneys, which can be surrounded by multiple structures such as bowel loops or vessels, and the size of the stones. Therapeutic options include open surgery, extracorporeal shock wave lithotripsy (ESWL), PCNL, laparoscopic or ultrasound-guided PCNL, and retrograde intrarenal surgery (RIRS) [6]. Historically, Eshghi et al. [7] were the first to report the successful application of laparoscopy-assisted PCNL for the treatment of pelvic kidney stones in 1985 [7].

Furthermore, such cases have traditionally been reported to be successful in multiple centers such as Mousavi-Bahar et al. [8] who utilized this method for three cases of the ectopic kidney which were treated successfully [8] as well as Maheshwari et al. [9] who described the same experience in a patient with horseshoe kidney and achieved a complete stone-free status in a single operation [9]. Similar to our case, they performed PCNL under laparoscopic and fluoroscopic guidance for direct access to the renal pelvis without extensive dissection or intracorporeal suturing [9] but our cases differ in terms of access as in this case the renal system was accessed through the middle calyx as opposed to the renal pelvis.

Moreover, given our experience with this case and the similar experiences of urologists over time, the use of laparoscopy-assisted PCNL appears to create a safe way of entering the abdomen and locating the ectopic kidney as well as provide visual guidance in puncture and dilatation all the while protecting the adjacent structures from harm; however, this procedure should only be undertaken by a urologist who has adequate prior experience in both laparoscopic surgery and PCNL procedures.

## Conclusions

Conclusively, this case demonstrates that laparoscopy-assisted PCNL is not only feasible but it is a safe, minimally invasive procedure for patients with ectopic kidneys presenting with large stones. Such cases highlight the advances made by the field of urology in the treatment of stone disease over time and emphasize that even challenging cases can be dealt with in a single setting with minimal hospital stay and complications.
